# Unusual Sequence of the Critical Magnetic Fields $H_{c1}$, $H_{c2}$, and $H_{c}$ in Multicomponent Superconductors

**DOI:** 10.1007/s10948-023-06664-8

**Published:** 2024-01-09

**Authors:** Yu.N. Ovchinnikov, D.V. Efremov

**Affiliations:** 1 Landau Institute for Theoretical Physics, RAS, Chernogolovka, Moscow District 142452 Russia; 2 Leibniz-Institut für Festkörper-und Werkstoffforschung Dresden, Dresden, Germany

**Keywords:** Multiband superconductors, Magnetic critical field

## Abstract

All superconductors in a magnetic field are characterized by three critical magnetic fields: lower critical $H_{c1}$, upper critical $H_{c2}$ and thermodynamic critical field $H_{c}$. Only two sets of inequalities $H_{c2}>H_c>H_{c1}$ or $H_{c1}>H_c>H_{c2}$ are possible in a single-component superconductor. Here, we report our study of the critical fields in multicomponent superconductors with two superconducting components in the framework of the Ginzburg-Landau functional. We derive the relationship between the phases of the components of the superconducting complex order parameter from the charge conservation law in explicit form and insert it into the Ginzburg-Landau functional. Using the modified Ginzburg-Landau equation, we acquire the single vortex state including the analytical expression for asymptotics. Also, we obtain the analytical form for the state in the upper critical field. We find that in some cases an unusual sequence of critical fields $H_{c1},H_{c2}>H_c$ can be realized in multicomponent superconductors.

## Introduction

The lower critical magnetic field $H_{c1}$ together with the upper critical field $H_{c2}$ and the thermodynamic critical field $H_c$ are the fundamental characteristics of superconductors, which describe the thermodynamics of a superconductor in an external magnetic field [1–4]. For one-component superconductors only two cases are possible: $H_{c1}>H_c>H_{c2}$ or $H_{c1}<H_c<H_{c2}$. The superconductors, in which the first inequality is satisfied, are called superconductors of the first kind. Correspondingly, if the second inequality is satisfied, superconductors are of the second kind. Recently, it was found that many superconductors such as Fe-based superconductors [5–8], MgB_2_ [9–13], Sr_2_RuO_4_ [14, 15], heavy fermion superconductors [16, 17], superconductivity at the interface between LaAlO_3_ and SrTiO_3_ [18] can not be described by a single-component order parameter. In this connection, a natural question arises, whether these two sequences of the inequalities exhaust all the possibilities in the case of multicomponent superconductors. This article aims to fill this gap.

Here, we show that a different sequence of critical magnetic fields can also be realized in a multicomponent superconductor. We use the conditional variation of the Ginzburg-Landau functional, i.e., the variation under the constraint proposed in [19]. In the presence of topological defects and some other cases, e.g., calculation of $H_{c2}$, the conditions $\delta F/\delta \phi _i = 0$ cannot be used for the derivation of a closed system of equations. Therefore the continuity equation $\text{ div }~ \textbf{j} =0$, which follows from the gradient in-variance of the Ginzburg-Landau functional, is used as an independent equation [20]. Resolving the continuity equation one gets a relation between $\left\{ \phi _i \right\}$ [20]. As a result only $N-1$ phase differences $\{\mu _{k} = \phi _1 - \phi _k\}$ can be considered as independent variables with one restriction mentioned above.

In this article, we imply the proposed scheme for a two-component superconductor. It allows to set up a closed system of equations for a state with a single vortex. For this state, we find analytically the asymptomatic behavior of the solutions at short and long distances from the vortex core and numerically at intermediate distances. We also obtain with the perturbation theory the equations for $H_{c2}$ for the two-component superconductor and compare the critical magnetic fields.

## The Functional

We start with a Ginzburg-Landau (GL) functional of a two-component superconductor in the form, in which the kinetic energy term is positively defined and diagonalized:

1 $$\begin{aligned} \mathcal{F}=& \int d^3r \left\{\sum _{i=1}^{2} \frac{\hbar ^2}{4m_i} \left| \left( \frac{\partial }{ \partial \textbf{r}} - \frac{2 i e }{\hbar c} \textbf{A} \right) \Psi _i \right| ^2 \right.\\&\qquad\left.- \left( U \hat{\Psi } \right) ^\dagger \hat{D} \left( U \hat{\Psi } \right) +\left( U_1 \hat{\Psi }^2 \right) ^\dagger \hat{D}_1 \left( U_1\hat{\Psi }^2 \right) \right\} \\&+ \frac{1}{8\pi } \int d^3 r \left( rot\textbf{A} -\textbf{H}_0\right) ^2. \end{aligned}$$

Here $\left\{ \hat{D }, \hat{D }_1 \right\}$ are diagonal matrices:

2 $$\begin{aligned} \hat{D } = \left( \begin{array}{cc} \ln \left( \frac{T_{c1}}{T}\right) &{} 0 \\ 0 &{}\ln \left( \frac{T_{c2}}{T}\right) \end{array} \right) , \quad \hat{D}_1 = \frac{1}{2}\left( \begin{array}{cc} b_1 &{} 0 \\ 0 &{}b_2 \end{array}\right) \end{aligned}$$

and $\left\{ U, U_1 \right\}$ are the Euler rotation matrices:

3 $$\begin{aligned} U = \left( \begin{array}{cc} \cos \theta &{} -\sin \theta \\ \sin \theta &{}\cos \theta \end{array} \right) , \quad U_1 = \left( \begin{array}{cc} \cos \theta _1 &{} -\sin \theta _1 \\ \sin \theta _1 &{} \cos \theta _1 \end{array}\right) \end{aligned}$$

with free parameters in the GL functional $\left\{ \theta ,\theta _1 \right\}$ and wave functions

4 $$\begin{aligned} \hat{\Psi } = \left( \begin{array}{c} \Psi _1 \\ \Psi _2 \end{array} \right) , \quad \hat{\Psi }^2 = \left( \begin{array}{cc} \Psi _1^2 \\ \Psi _2^2 \end{array}\right) . \end{aligned}$$

A multi-component superconductor may possess a phase shift between the components of the order parameter, which is different from $\{0, \pi \}$ already in a zero external magnetic field. In a such superconductor, the time-reversal symmetry is broken. Superconductors of this kind will be referred to in the text as superconductors with broken time-reversal symmetry (BTRS) or BTRS superconductors (for classification of classes of superconductors see [17, 21]). Both of the cases, with time-reversal symmetry and with broken time-reversal symmetry can be described in the framework of the Ginzburg-Landau functional. For considering below a two-component superconductor it means that two modulus of the order parameters, phase difference, and the vector potential $\textbf{A}$ can be considered as independent variables. Variation of the Ginzburg-Landau functional in these variables leads to a set of four differential equations. The solution of these equations gives the state of the superconductor in an external magnetic field.

Since the system with a single vortex is a rotational invariant, it is convenient to use the cylindrical system of coordinates ($\textbf{r} = (\rho \cos \phi , \rho \sin \phi ,z)$). Then, we take the components of the wave function $\Psi _i = \Psi _i(\rho ,\phi )$ in the form:

5 $$\begin{aligned} \Psi _i = |\Psi _i| e^{i \chi _i}, \quad \chi _i = \phi +\tilde{\phi }_i, \end{aligned}$$

where $\phi$ is the polar angle and $\tilde{\phi }_i = \tilde{\phi }_i(\rho )$ are functions depending on $\rho$. From Eq. (5) one gets

6 $$\begin{aligned} \partial _-\Psi _i =&\; \textbf{e}_\rho \left\{ \frac{\partial |\Psi _i|}{\partial \rho } + i {|\Psi _i|}\left( \frac{\partial \tilde{\phi }_i}{\partial \rho } - \frac{2e}{\hbar c} A_\rho \right) \right\} e^{i\chi _i } \\&+ \textbf{e}_\phi i |\Psi _i| \left\{ \frac{1}{\rho } - \frac{2e}{\hbar c} A_\phi \right\} e^{i\chi _i } , \end{aligned}$$

where

7 $$\begin{aligned} \textbf{A} &= \textbf{e}_\rho A_\rho + \textbf{e}_\phi A_\phi , \frac{2e}{\hbar c } A_\rho = \frac{\partial \Phi }{\partial \rho },\\ \textbf{e}_\rho &= (\cos \phi , \sin \phi ), \textbf{e}_\phi = (-\sin \phi , \cos \phi ). \end{aligned}$$

The current density in the single vortex state is

8 $$\begin{aligned} \textbf{j} = e \hbar \sum _{i = 1}^{2} \frac{|\Psi _i|^2}{m_i} \left[ \textbf{e}_\rho \frac{ \partial ( \tilde{\phi }_i -\Phi )}{\partial \rho } + \textbf{e}_\phi \left( \frac{1}{\rho } - \frac{2e}{\hbar c} A_\phi \right) \right] . \end{aligned}$$

From the symmetry considerations, the radial part of the current vanishes. Hence, from Eq. (8), we get

9 $$\begin{aligned} \frac{1}{m_1} |\Psi _1|^2 \frac{ \partial ( \tilde{\phi }_1 - \Phi )}{\partial \rho } + \frac{1}{m_2} |\Psi _2|^2 \frac{ \partial (\tilde{\phi }_2 - \Phi )}{\partial \rho }=0. \end{aligned}$$

To resolve Eq. (9), we introduce a new function $\mu (\rho )$:

10 $$\begin{aligned} \mu (\rho ) = \tilde{\phi }_1 - \tilde{\phi }_2 . \end{aligned}$$

with $\mu (\rho )$ being a solution of

11 $$\begin{aligned} \frac{\partial \mu }{\partial \rho } = \left( 1 + \frac{m_2}{m_1} \frac{|\Psi _1|^2}{|\Psi _2|^2} \right) \frac{\partial }{\partial \rho } (\tilde{\phi }_1 - \Phi ). \end{aligned}$$

Here, we would like to note that the equations obtained by variations of the functional over $\tilde{\phi }_i$ cannot be used as independent equations to determine $\tilde{\phi }_i$ anymore due to the above constraint. Resoling Eq. (11), we get

12 $$\begin{aligned} \frac{\partial (\tilde{\phi }_1-\Phi )}{\partial \rho } = \frac{\partial \mu }{\partial \rho }\Gamma , \quad \frac{\partial (\tilde{\phi }_2-\Phi )}{\partial \rho } = \frac{\partial \mu }{\partial \rho }(\Gamma -1). \end{aligned}$$

These equations are the key point of the solution to the problem under consideration. Now, we can rewrite the functional Eq. (1) in the form:

13 $$\begin{aligned} \begin{aligned} \tilde{\mathcal{F}}&= \int d^3 r \left\{ \frac{\hbar ^2}{4m_1} \left[ \left( \frac{\partial |\Psi _1|}{\partial \rho } \right) ^2 + |\Psi _1 |^2 \left( \left( \frac{\partial (\tilde{\phi }_1 - \Phi )}{\partial \rho } \right) ^2 +\left( \frac{1}{\rho } - \frac{2e}{\hbar c} A_\phi \right) ^2 \right) \right] \right. \\ {}&+ \frac{\hbar ^2}{4m_2} \left[ \left( \frac{\partial |\Psi _2|}{\partial \rho } \right) ^2 + |\Psi _2 |^2 \left( \left( \frac{\partial (\tilde{\phi }_2 - \Phi )}{\partial \rho } \right) ^2 +\left( \frac{1}{\rho } - \frac{2e}{\hbar c} A_\phi \right) ^2 \right) \right] \\ {}&+ \left. \left( U_1 \left( \begin{array}{c} |\Psi _1|^2 e^{2i \tilde{\phi }_1} \\ |\Psi _2|^2 e^{2i \tilde{\phi }_2} \end{array} \right) \right) ^\dagger \hat{D}_1 \left( U_1 \left( \begin{array}{c} |\Psi _1|^2 e^{2i \tilde{\phi }_1} \\ |\Psi _2|^2 e^{2i \tilde{\phi }_2} \end{array} \right) \right) - \left( U \left( \begin{array}{c} |\Psi _1| e^{i \tilde{\phi }_1} \\ |\Psi _2| e^{i \tilde{\phi }_2} \end{array} \right) \right) ^\dagger \hat{D} \left( U \left( \begin{array}{c} |\Psi _1| e^{i \tilde{\phi }_1} \\ |\Psi _2| e^{i \tilde{\phi }_2} \end{array} \right) \right) \right\} \\&+ \frac{1}{8\pi } \int d^3r (rot(\textbf{e}_\phi A_\phi ) - H_0)^2 \end{aligned} \end{aligned}$$

If Eqs. (9, 10 and 11) are satisfied, minimization of functional $\tilde{ \mathcal F}$ produces for functions $\left\{ |\Psi _1|, |\Psi _2|, A_\phi , \mu \right\}$ four equations. Minimizing the functional Eq. (13), we find the equations for $\{|\Psi _1|, |\Psi _2|\}$:

14 $$\begin{aligned} \begin{aligned} \frac{\hbar ^2 }{2m_1}&\left[ - \frac{1}{\rho }\frac{\partial }{\partial \rho } \left( \rho \frac{\partial }{\partial \rho }\right) |\Psi _1| + \left( \Gamma ^2 \left( \frac{\partial \mu }{\partial \rho }\right) ^2 + \left( \frac{1}{\rho } - \frac{2e}{\hbar c} A_\phi \right) ^2 \right) |\Psi _1| \right] \\ {}&+ 2 |\Psi _1|^3 (b_1 \cos ^2\theta _1 + b_2 \sin ^2 \theta _1 ) - \sin (2 \theta _1) |\Psi _1||\Psi _2|^2 (b_1-b_2) \cos (2\mu ) \\ {}&- 2 |\Psi _1|\left( \cos ^2 \theta \ln \left( \frac{T_{c1}}{T}\right) +\sin ^2 \theta \ln \left( \frac{T_{c2}}{T}\right) \right) + \sin (2\theta ) |\Psi _2| \ln \left( \frac{T_{c1}}{T_{c2}}\right) \cos \mu = 0 \end{aligned} \end{aligned}$$

and

15 $$\begin{aligned} \begin{aligned} \frac{\hbar ^2 }{2m_2}&\left[ - \frac{1}{\rho }\frac{\partial }{\partial \rho } \left( \rho \frac{\partial }{\partial \rho }\right) |\Psi _2| + \left( (\Gamma -1)^2 \left( \frac{\partial \mu }{\partial \rho }\right) ^2 + \left( \frac{1}{\rho } - \frac{2e}{\hbar c} A_\phi \right) ^2 \right) |\Psi _2|\right] \\ {}&+ 2 |\Psi _2|^3 (b_1 \sin ^2\theta _1 + b_2 \cos ^2 \theta _1 ) - \sin (2 \theta _1) |\Psi _1|^2|\Psi _2| (b_1-b_2) \cos (2\mu ) \\ {}&- 2 |\Psi _2|\left( \sin ^2 \theta \ln \left( \frac{T_{c1}}{T}\right) +\cos ^2 \theta \ln \left( \frac{T_{c2}}{T}\right) \right) + \sin (2\theta ) |\Psi _1| \ln \left( \frac{T_{c1}}{T_{c2}}\right) \cos \mu = 0 , \end{aligned} \end{aligned}$$

where

16 $$\begin{aligned} \Gamma = \left( 1 + \frac{m_2}{m_1} \frac{|\Psi _1|^2}{|\Psi _2|^2}\right) ^{-1}. \end{aligned}$$

Further, variation of $\tilde{\mathcal{F}}$ with respect to $\mu$ gives

17 $$\begin{aligned} \begin{aligned} \frac{\hbar ^2}{2m_1}&\left( - \frac{1}{\rho } \frac{\partial }{\partial \rho } \left( \rho |\Psi _1|^2 \Gamma ^2 \frac{\partial \mu }{\partial \rho } \right) \right) + \frac{\hbar ^2}{2m_2} \left( - \frac{1}{\rho } \frac{\partial }{\partial \rho } \left( \rho |\Psi _2|^2 (\Gamma -1)^2 \frac{\partial \mu }{\partial \rho } \right) \right) \\ {}&+ \sin (2\theta _1)|\Psi _1|^2 |\Psi _2|^2 (b_1 -b_2) \sin (2 \mu ) - \sin (2\theta ) |\Psi _1| |\Psi _2| \ln \left( \frac{T_{c1}}{T_{c2}} \right) \sin \mu = 0. \end{aligned} \end{aligned}$$

The gauge is determined by the Maxwell equation for the vector potential $A_\phi$:

18 $$\begin{aligned} \begin{aligned} -\frac{1}{\rho } \frac{\partial }{\partial \rho } \left( \rho \frac{\partial A_\phi }{\partial \rho } \right) &+ \frac{8\pi e^2}{c^2} \left( \frac{1}{m_1}|\Psi _1|^2+\frac{1}{m_2}|\Psi _2|^2 \right) A_\phi+ \frac{1}{\rho ^2} A_\phi \\&= \frac{4\pi e\hbar }{c} \left( \frac{1}{m_1}|\Psi _1|^2+\frac{1}{m_2}|\Psi _2|^2 \right) \frac{1}{\rho }. \end{aligned} \end{aligned}$$

and the boundary conditions. At $\rho \rightarrow \infty$ vector potential $A_\phi$ tends to

19 $$\begin{aligned} A_\phi \rightarrow \frac{\hbar c }{2e} \frac{1}{\rho }. \end{aligned}$$

As a result, we obtain the following quantization rule for one flux:

20 $$\begin{aligned} \int d^2r \textbf{H}(\rho ) = \frac{\pi \hbar c}{e}=\Phi _0, \end{aligned}$$

where $\Phi _0$ is the flux quantum. The effective penetration depth is

21 $$\begin{aligned} \lambda ^{-2} = \frac{8\pi e^2}{c^2} \left( \frac{1}{m_1} |\Psi _1|^2+\frac{1}{m_2} |\Psi _2|^2 \right) _{\rho \rightarrow \infty }. \end{aligned}$$

Using Eq. (20), we can obtain the next expression for the first magnetic critical field $H_{c1}$.

22 $$\begin{aligned} \frac{H_{c1}}{4\pi } \Phi _0 = \int d^2r \frac{H^2(\rho )}{8\pi } + \int d^2r \left( f_1^{(1)} - f_1^{(0)}\right) . \end{aligned}$$

**Fig. 1:**
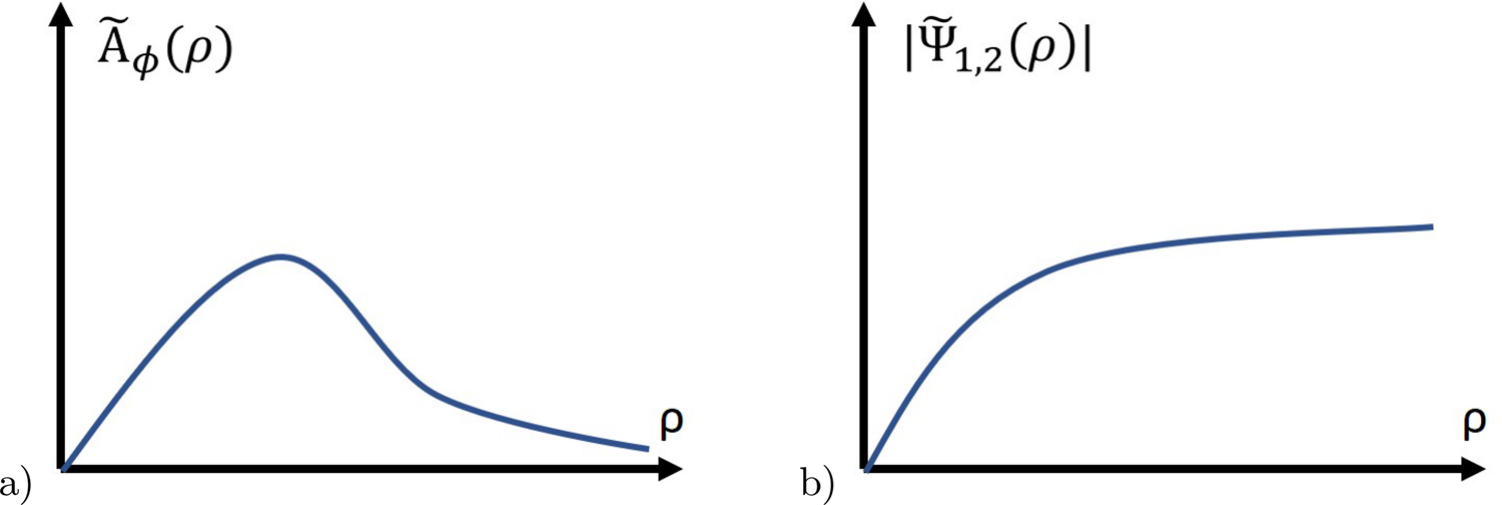
Schematic $\rho$-dependence of $\tilde{A}_\phi (\rho )$ and $\tilde{\Phi }_\{1,2\} (\rho )$

Here $f_1^{(0,1)}$ are the density of the condensate energy in the ground state and in the state with a single vortex:

23 $$\begin{aligned} \begin{aligned} f_1^{(1)}&= \frac{\hbar ^2}{4m_1} \left[ \left( \frac{\partial |\Psi _1|}{\partial \rho } \right) ^2 + |\Psi _1|^2 \left( \Gamma ^2 \left( \frac{\partial \mu }{\partial \rho }\right) ^2 + \left( \frac{1}{\rho } - \frac{2e}{\hbar c} A_\phi \right) ^2 \right) \right] \\&+\frac{\hbar ^2}{4m_2} \left[ \left( \frac{\partial |\Psi _2|}{\partial \rho } \right) ^2 + |\Psi _2|^2 \left( (1-\Gamma )^2 \left( \frac{\partial \mu }{\partial \rho }\right) ^2 + \left( \frac{1}{\rho } - \frac{2e}{\hbar c} A_\phi \right) ^2 \right) \right] \\&+ \frac{1}{2} [ |\Psi _1|^4(b_1 - \sin ^2\theta _1 (b_1 - b_2) ) + |\Psi _2|^4(b_1 - \cos ^2\theta _1 (b_1-b_2) ) -\sin (2\theta _1) |\Psi _1|^2 |\Psi _2|^2 (b_1 - b_2) \cos (2\mu ) ] \\&- |\Psi _1|^2\!\! \left( \cos ^2\theta \ln \!\!\left( \frac{T_{c1}}{T}\right) +\sin ^2\theta \ln \!\!\left( \frac{T_{c2}}{T}\right) \right) - |\Psi _2|^2\!\! \left( \sin ^2\theta \ln \!\!\left( \frac{T_{c1}}{T}\right) +\cos ^2\theta \ln \!\!\left( \frac{T_{c2}}{T}\right) \right) \\&+ \sin (2\theta ) |\Psi _1||\Psi _2|\ln \left( \frac{T_{c1}}{T_{c2}}\right) \cos \mu \end{aligned} \end{aligned}$$

and

24 $$\begin{aligned} \begin{aligned} f_1^{(0)}&= \frac{1}{2} [ |\Psi _1^{(0)}|^4(b_1 - \sin ^2\theta _1 (b_1 - b_2) ) + |\Psi _2^{(0)}|^4(b_1 - \cos ^2\theta _1 (b_1-b_2) ) \\ {}&-\sin (2\theta _1) |\Psi ^{(0)}_1|^2 |\Psi ^{(0)}_2|^2 (b_1 - b_2) \cos (2(\phi ^{(0)}_2-\phi ^{(0)}_1)) ]\\&- \left[ |\Psi _1^{(0)}|^2\!\! \left( \cos ^2\theta \ln \!\!\left( \frac{T_{c1}}{T}\right) +\sin ^2\theta \ln \!\!\left( \frac{T_{c2}}{T}\right) \right) + |\Psi _2^{(0)}|^2\!\! \left( \sin ^2\theta \ln \!\!\left( \frac{T_{c1}}{T}\right) +\cos ^2\theta \ln \!\!\left( \frac{T_{c2}}{T}\right) \right) \right] \\&+ \sin (2\theta ) |\Psi _1^{(0)}||\Psi _2^{(0)}|\ln \left( \frac{T_{c1}}{T_{c2}}\right) \cos (\phi ^{(0)}_1-\phi ^{(0)}_2), \end{aligned} \end{aligned}$$

where the functions $\Psi _{1,2}^{(0)}$ are the values of the correspondent functions in the ground state.

**Fig. 2:**
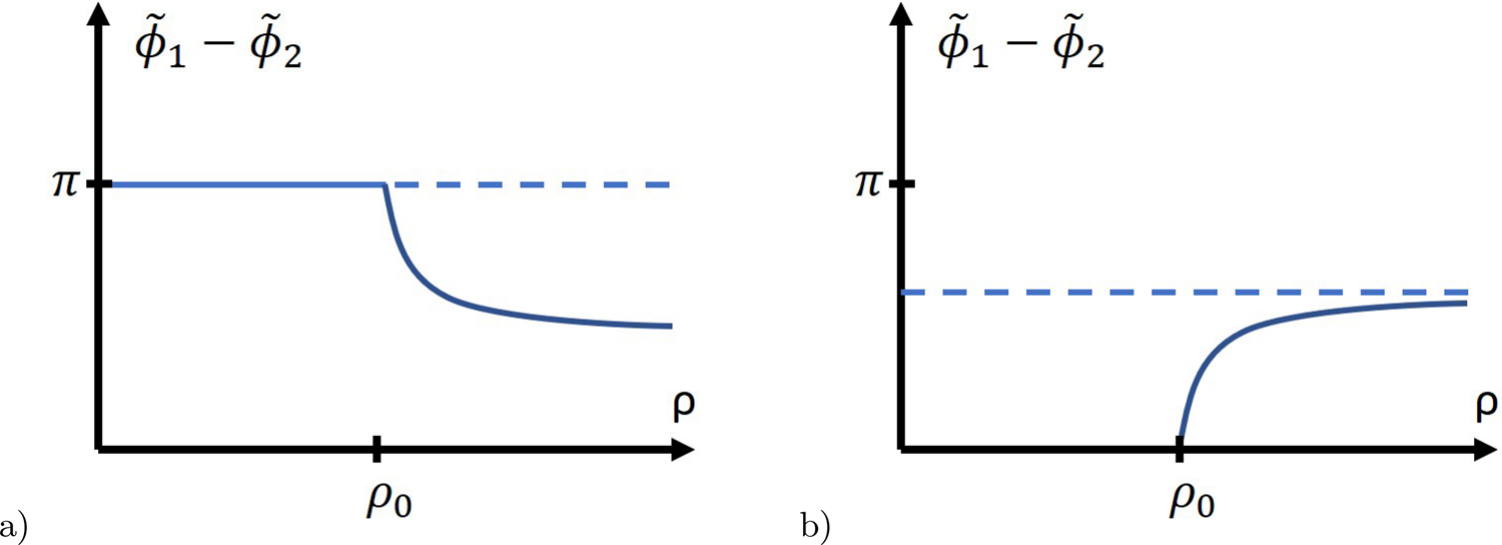
Two possible $\rho$ dependencies of $\tilde{\phi }_1 - \tilde{\phi }_2$. More details see in the text

In the dimensionless variables, we obtain (see Appendix A):

25 $$\begin{aligned} \begin{aligned} \tilde{H}_{c1}&= \frac{1}{2}\int _{0}^{+\infty } dt_0 t_0 \tilde{H}^2 \\&+ \left( \frac{4\pi e^2 \gamma ^2}{m_1 c^2} |\Psi _1|_{inf}^2 \right) \int _{0}^{+\infty } dt_0 t_0 \left\{ \left( \frac{\partial |\tilde{\Psi }_1|}{\partial t_0}\right) ^2 + |\tilde{\Psi }_1|^2 \left( \Gamma ^2 \left( \frac{\partial \mu }{\partial t_0}\right) ^2 +\frac{1}{t_0^2}(1 -\tilde{A}t_0)^2 \right) \right. \\ {}&+ \left. \frac{m_1}{m_2} \frac{|\Psi _2|^2_{inf}}{|\Psi _1|^2_{inf}} \left[ \left( \frac{\partial |\tilde{\Psi }_2|}{\partial t_0}\right) ^2 + |\tilde{\Psi }_2|^2 \left( (1 - \Gamma )^2\left( \frac{\partial \mu }{\partial t_0}\right) ^2 + \frac{1}{t_0^2} (1 - \tilde{A}t_0)^2 \right) \right] \right\} \\ {}&+ \left( \frac{4\pi e^2 \gamma ^2}{m_1 c^2} |\Psi _1|_{inf}^2 \right) (b_1 |\Psi _1|_{inf}^2)\int _{0}^{+\infty } dt_0 t_0 \left\{ (|\tilde{\Psi }_1|^4-1) \left( \cos ^2 \theta _1 + \frac{b_2}{b_1}\sin ^2\theta _1 \right) \right. \\&+ \left. \frac{|\Psi _2|_{inf}^4}{|\Psi _1|_{inf}^4} (|\tilde{\Psi }_2|^4-1)\left( \sin ^2 \theta _1 + \frac{b_2 }{b_1} \cos ^2 \theta _1\right) \right\} \\&-\left( \frac{4\pi e^2 \gamma ^2}{m_1 c^2} |\Psi _1|_{inf}^2 \right) (b_1 |\Psi _2|_{inf}^2) \left( 1- \frac{b_2}{b_1} \right) \sin (2 \theta _1) \int _{0}^{+\infty } dt_0 t_0 \left\{ |\tilde{\Psi }_1 |^2 |\tilde{\Psi }_2 |^2 \cos (2\mu ) - \cos (2\mu _{inf}) \right\} \\&- \left( \frac{8\pi e^2 \gamma ^2}{m_1 c^2} |\Psi _1|_{inf}^2 \right) \int _{0}^{+\infty } dt_0 t_0 \left\{ \left( \cos ^2 \theta \ln \left( \frac{T_{c1}}{T} \right) + \sin ^2 \theta \ln \left( \frac{T_{c2}}{T} \right) \right) (|\tilde{\Psi }_1|^2-1) \right. \\&+ \left. \left. \frac{|\Psi _2|_{inf}^2}{|\Psi _1|_{inf}^2} \left( \sin ^2 \theta \ln \left( \frac{T_{c1}}{T} \right) + \cos ^2 \theta \ln \left( \frac{T_{c2}}{T} \right) \right) (|\tilde{\Psi }_2|^2-1) \right. \right. \\&- \left. \sin (2\theta ) \frac{|\Psi _2|_{inf}}{|\Psi _1|_{inf}} \ln \left( \frac{T_{c1}}{T_{c2}} \right) (|\tilde{\Psi }_1||\tilde{\Psi }_2|\cos (\mu ) - \cos (\mu _{inf})) \right\} \end{aligned} \end{aligned}$$

The results of the numerical calculations of the first and second critical magnetic fields $H_{c1}, H_{c2},$ and also the thermodynamic critical field $\tilde{H}_{c}$ are given in Table 1. Note, that dependence of $\tilde{H}_{c}$ from $\theta$ is weak. An increase of $\theta$ leads to the evolution of the superconductivity so that $H_{c1}$ and $H_c$ cross with the formation of a nontrivial transition region.

The ground state without vortices can be of two types. The first type is with preserved time-reversal symmetry $\sin (\tilde{\phi }_1 - \tilde{\phi }_2)=0$. The second type is the state with broken time-reversal symmetry, which has the solution with $\sin (\tilde{\phi }_1 - \tilde{\phi }_2) \ne 0$. The first case is trivial. In the second case a separate point can exist $\{\rho =\rho _0\}$ (see Fig. 2). Below this point in the single vortex solution, $|\Psi _{1,2}|$ depend on $\rho$, but $\tilde{\phi }_1 - \tilde{\phi }_2 = \{0, \pi \}$. As a result Eqs. (13, 14, 16 and 18) shrink to three equations for $\{ |\Psi _1|, |\Psi _2|, A_\phi \}$ as in the case with preserved time-reversal symmetry.

Solving the set of equations, one gets the asymptotics:

26 $$\begin{aligned} A_\phi = \frac{H(0)}{2} \rho \text{ at } \rho \ll \lambda , \text{ and } A_\phi = \frac{\hbar c}{2e} \frac{1}{\rho } \text{ at } \rho \gg \lambda , \end{aligned}$$

where *H*(0) is the value of the magnetic field at the center of the vortex core. The functions $\{|\Psi _{1,2}|\}$ are proportional to $\rho$ at the distances smaller than the correlation length and approaches with an exponential decay to a constant at large $\rho$. Qualitative $\rho$-dependence of $A_\phi$,$\tilde{\phi }_1 - \tilde{\phi }_2$ and $|\Psi _{1,2}|$ are presented in Fig. 1a and b.

**Fig. 3:**
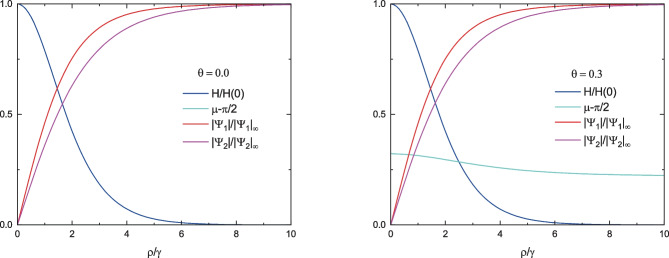
Normalized magnetic field $H/H_0$, phase $\phi$, and wave functions $|\Psi _1|/|\Psi _1|\infty$. as function of $\rho /\rho _0$. The parameters are $\gamma ^2 = \frac{\hbar ^2}{2m_1}$, $\mu = \tilde{\phi }_1 - \tilde{\phi }_2$

Using Eq. (17) one can estimate the value of parameter $\rho _0$:

27 $$\begin{aligned} \left\{ \frac{m_2}{m_1} \frac{\partial }{\partial \rho } \left( \rho |\Psi _1|^2 \Gamma ^2\right) + \frac{\partial }{\partial \rho } \left( \rho |\Psi _2|^2 (1-\Gamma )^2\right) \right\} _{\rho = (\rho _0)_+} = 0 \end{aligned}$$

The value of the slope $\left( \frac{\partial \mu }{\partial \rho }\right) _{\rho = (\rho )_+}$ is a free parameter. Its value is fixed by the boundary conditions at infinity. As a result, we get a weak singularity in the functions $\{|\Psi _1|,|\Psi _2|\}$ since the functions themselves and their first derivatives continue at this point.

At large subspace of the intrinsic parameters, the value of $\rho _0$ is located in the nonphysical region ($\rho < 0$). The intrinsic parameters, used by us for numerical calculations belong to such subspace. The simplest situation for calculations arises for $\theta = 0$. In such case the solution of Eq. (17) is

28 $$\begin{aligned} \mu (\rho ) = \pm \frac{\pi }{2}. \end{aligned}$$

For parameters:

29 $$\begin{aligned} m_1 =&\; 2 m_2, b_2=2b_1, \quad b_1 = 1.5 \cdot 10^{-5} G^{-2}, \\&T_{c1}/T = 1.2,\quad T_{c2}/T = 1.1 \end{aligned}$$

and

$$\begin{aligned} \frac{\hbar ^2}{4m_1} &= 2.7773 \cdot 10^{-11} cm^2, \\ \theta _1 &= 0.5, \theta = \{0,0.1,0.3\} \end{aligned}$$

the dependencies $|\tilde{\Psi }_{1,2}|$,$\tilde{B}(\rho )$ and $(\tilde{\phi }_1 -\tilde{\phi }_2)_{\rho }$ for $\theta =0$ and $\theta =0.3$ are given in Fig. 2a. For the numerical calculations, we have used dimensionless equations. The details of the numerical calculations are presented in Appendices A-G.

From Eqs. (13-15), we obtain the next values of $\{|\Psi _1|,|\Psi _2|,\tilde{\phi }_1 - \tilde{\phi }_2\}$ at $\rho \rightarrow \infty$ in the state with broken time-reversal symmetry:

30 $$\begin{aligned} \cos (\tilde{\phi }_1 - \tilde{\phi }_2)|_\infty = \frac{\sin (2\theta ) \ln (T_{c1}/T_{c2}) }{2|\Psi _1||\Psi _2| \sin (2\theta _1)(b_1 -b_2)}, \end{aligned}$$

and

31 $$\begin{aligned} \begin{aligned} |\Psi _1|^2 (b_1\cos ^2\theta _1 + b_2 \sin ^2 \theta _1)&+ \frac{1}{2} \sin (2\theta _1) |\Psi _{2}|^2 (b_1-b_2) \\&= \left( \cos ^2 \theta \ln \left( \frac{T_{c1}}{T}\right) +\sin ^2 \theta \ln \left( \frac{T_{c2}}{T}\right) \right) \end{aligned} \end{aligned}$$

32 $$\begin{aligned} \begin{aligned} |\Psi _2|^2 (b_2\cos ^2\theta _1 + b_1 \sin ^2 \theta _1)&+ \frac{1}{2} \sin (2\theta _1) |\Psi _{1}|^2 (b_1-b_2) \\&= \left( \cos ^2 \theta \ln \left( \frac{T_{c2}}{T}\right) +\sin ^2 \theta \ln \left( \frac{T_{c1}}{T}\right) \right) \end{aligned} \end{aligned}$$

The considered state corresponds to the minima of the free energy functional provided the following inequality is satisfied:

$$\begin{aligned} \frac{\sin ^2(2\theta ) \ln ^2(T_{c1}/T_{c2})}{4\sin ^2(2\theta _1) (b_1- b_2)^2}<|\Psi _{1}|^2|\Psi _{2}|^2. \end{aligned}$$

Obviously, for this case, Eqs. (30)-(32) give a single solution and, therefore, they describe the global minimum.

In this case, the vector potential $(A_\phi - (\hbar c)/(2e\rho ))$ decays exponentially at infinity as $\propto \exp (-\rho /\lambda ){ /\sqrt{\rho }}$, where the parameter $\lambda$ is given by the Eq. (21). The three quantity $\{\delta \mu , \delta |\Psi _1|, \delta |\Psi _2|\}$ of the difference of the correspondent values from that at $\rho \rightarrow \infty$ decay exponentially at large distances as well:

33 $$\begin{aligned} \left( \begin{array}{c} \delta \mu \\ \delta |\Psi _1| \\ \delta |\Psi _2| \end{array} \right) =&\; C_1 \exp (-\kappa _1^{(1)}\rho ){ \frac{1}{\sqrt{\rho }}}\textbf{f}_1 + C_2 \exp (-\kappa _1^{(2)}\rho ){ \frac{1}{\sqrt{\rho }}}\textbf{f}_2 \\&+ C_3 \exp (-\kappa _1^{(3)}\rho ){ \frac{1}{\sqrt{\rho }}} \textbf{f}_3, \end{aligned}$$

where the $C_i$ with $i=1,2,3$ are some coefficients, while $\kappa _1^{(i)}$ and $\textbf{f}_i$ are eigenvalues and eigenvectors of the next system:

34 $$\begin{aligned} \tilde{\tilde{D}} \left( \begin{array}{c} \delta \mu \\ \delta |\Psi _1| \\ \delta |\Psi _2| \end{array} \right) =0. \end{aligned}$$

Here $\tilde{\tilde{ D}}$ is a Hermitian operator with the following elements:

35 $$\begin{aligned} \begin{aligned} \tilde{\tilde{ D}}_{11}&= - \kappa _1^2 \left( \frac{\hbar ^2|\Psi _{1}^2| \Gamma ^2}{2m_1} + \frac{\hbar ^2|\Psi _{2}^2| (1 -\Gamma )^2}{2m_2} \right) -\sin (2\theta ) |\Psi _{1}||\Psi _{2}|\ln \left( \frac{T_{c1}}{T_{c2}}\right) \cos \mu + 2\sin (2\theta _1) |\Psi _{1}|^2 |\Psi _{2}|^2(b_1 -b_2)\cos (2\mu ) \\ \tilde{\tilde{ D}}_{22}&= - \kappa _1^2 \frac{\hbar ^2}{2m_1} + 6|\Psi _{1}|^2 (b_1 \cos ^2 \theta _1 + b_2 \sin ^2 \theta _1 ) - \sin (2\theta _1 )(b_1 -b_2)|\Psi _{2}|^2 \cos (2 \mu ) - 2 \left( \!\!\cos ^2\theta \ln \left( \frac{T_{c1}}{T}\right) +\sin ^2\theta \ln \left( \frac{T_{c2}}{T}\right) \!\!\right) , \\ \tilde{\tilde{ D}}_{33}&= - \kappa _1^2 \frac{\hbar ^2}{2m_2} + 6|\Psi _{2}|^2 (b_2 \cos ^2 \theta _1 + b_1 \sin ^2 \theta _1 ) - \sin (2\theta _1 )(b_1 -b_2)|\Psi _{1}|^2 \cos (2 \mu )- 2 \left( \!\!\sin ^2\theta \ln \left( \frac{T_{c1}}{T}\right) +\cos ^2\theta \ln \left( \frac{T_{c2}}{T}\right) \!\!\right) , \\ \tilde{\tilde{ D}}_{12}&= \tilde{\tilde{ D}}_{21} = - \sin (2\theta ) |\Psi _{2}|\sin \mu \ln \left( \frac{T_{c1}}{T_{c2}}\right) + 2\sin (2\theta _1 )(b_1 -b_2)|\Psi _{1}| |\Psi _{2}|^2\sin (2 \mu ) \\ \tilde{\tilde{ D}}_{13}&= \tilde{\tilde{ D}}_{31} = - \sin (2\theta ) |\Psi _{1}|\sin \mu \ln \left( \frac{T_{c1}}{T_{c2}}\right) + 2\sin (2\theta _1 )(b_1 -b_2)|\Psi _{1}|^2 |\Psi _{2}|\sin (2 \mu ) \\ \tilde{\tilde{ D}}_{23}&= \tilde{\tilde{ D}}_{32} = -2 \sin (2\theta _1) |\Psi _{1}||\Psi _{2}|(b_1-b_2)\cos (2\mu ) + \sin (2\theta ) \ln \left( \frac{T_{c1}}{T_{c2}}\right) \cos \mu \end{aligned} \end{aligned}$$

By the correct boundary conditions, the solution at large distances tends to the that given by Eq. (33). The correspondent free parameters for Eqs. (14, 15 and 18) are the slopes at $\rho = 0$ of $|\tilde{\Psi }_{1}|$, $|\tilde{\Psi }_{2}|$ and $\tilde{A}_\phi$. For $\tilde{\mu }$ at $\rho =0$ the initial condition is $\mu (0)$ if $\rho _0$ does not exists, and Eq. (27) otherwise. At this point, we note that at large distance $|\Psi _{1}|$, $|\Psi _{2}|$ and $\mu$ decay with the same exponent due to the coupling between the components.

## Critical Field $H_{c2}$

At the critical point $H_{c2}$ the order parameters can be found with the following Ansatz:

36 $$\begin{aligned} \left( \begin{array}{c} |\Psi _{1}| \\ |\Psi _{2}| \end{array} \right) = \Psi \left( \begin{array}{c} C_{1} \\ C_{2} \end{array} \right) \end{aligned}$$

where $\Psi$ is the solution of the equation [1]:

37 $$\begin{aligned} -\partial _-^2 \Psi = \eta \Psi , \textbf{A} = (0,Hx,0), \textbf{H} = (0,0,H) \end{aligned}$$

and $C_1$ and $C_2$ are constants. The solution of Eq. (37) is

38 $$\begin{aligned} \Psi = \exp \left\{ -\frac{eH}{\hbar c}(x -x_0)^2 + \frac{2ieH}{\hbar c}x_0y \right\} \end{aligned}$$

with $\eta = 2eH/\hbar c$ and $x_0$ being a free parameter.

For $\eta$, we obtain the following quadratic equation

39 $$\begin{aligned} \det \left( \begin{array}{cc} \frac{\hbar ^2}{ 4m_1} \eta - \left( \cos ^2 \theta \ln \left( \frac{T_{c1}}{T}\right) +\sin ^2 \theta \ln \left( \frac{T_{c2}}{T}\right) \right) &{} \frac{1}{2}\ln \left( \frac{T_{c1}}{T_{c2}}\right) \sin (2\theta ) \\ \frac{1}{2}\ln \left( \frac{T_{c1}}{T_{c2}}\right) \sin (2\theta ) &{} \frac{\hbar ^2}{ 4m_2} \eta - \left( \sin ^2 \theta \ln \left( \frac{T_{c1}}{T}\right) +\cos ^2 \theta \ln \left( \frac{T_{c2}}{T}\right) \right) \end{array} \right) = 0. \end{aligned}$$

Solving these equations, we get $H_{c2}$:

40 $$\begin{aligned} \begin{aligned}&\frac{\hbar e}{c}H_{c2} = m_1 \left( \cos ^2 \theta \ln \frac{T_{c1}}{T} +\sin ^2 \theta \ln \frac{T_{c2}}{T} \right) + m_2 \left( \sin ^2 \theta \ln \frac{T_{c1}}{T} +\cos ^2 \theta \ln \frac{T_{c2}}{T} \right) \\ {}&+ \!\left[ \! \left( m_1 \!\left( \! \cos ^2 \theta \ln \frac{T_{c1}}{T} +\sin ^2 \theta \ln \frac{T_{c2}}{T} \right) - m_2 \!\left( \sin ^2 \theta \ln \frac{T_{c1}}{T} +\cos ^2 \theta \ln \frac{T_{c2}}{T} \right) \right) ^2 \!+\!\! m_1 m_2 \ln ^2 \left( \frac{T_{c1}}{T_{c2}} \right) \sin ^2 (2 \theta ) \right] ^{1/2} \end{aligned} \end{aligned}$$

The numerical results are

41 $$\begin{aligned} H_{c2} = \frac{\hbar c}{ 2 e \gamma ^2} \tilde{ H}_{c2} \end{aligned}$$

and

42 $$\begin{aligned} \theta = 0: \tilde{H}_{c2} = 2\ln 1.2 = 0.36464 \end{aligned}$$

43 $$\begin{aligned} \theta = 0.3: \tilde{H}_{c2} = 0.3542472. \end{aligned}$$

In both cases, we obtain that the critical fields $H_{c1}$ and $H_{c2}$ are larger than the thermodynamic $H_c$. Hence, the transition to the vortex state takes place at the external field equal $H_{c2}$. However, the transition to the homogeneous case happens at $H = H_c$ as a transition of the first order accompanied by a jump in the magnetic moment value. In the region $H_{c2}>H>H_{c}$ a cascade of transitions with change of the structure of the vortex state is possible [22].

## Conclusions

We considered a single vortex state and the first critical magnetic field $H_{c1}$ in a multicomponent superconductor with *N* components in the framework of the Ginzburg-Landau functional. It has been shown that the problem can be reduced to solving a system of $2N-1$ ordinary differential equations if in the ground state, the phase shift between the component of the complex order parameter is 0 or $\pi$ at zero external magnetic field. Otherwise, it consists of 2*N* equations. At $\rho \rightarrow \infty$ the phase difference between the components of the order parameter $\mu _k=\phi _1 - \phi _k$ does not tend to $0, \pi$. And the $\mu$ can reach the values $0, \pi$ only at finite $\rho = \rho _0$ and for $\rho <\rho _0$ the solution $\mu = 0, \pi$ is realized (see Fig. 2).

In a single-component superconductor in a magnetic field, the state is determined by the Ginzburg-Landau parameter $\kappa ^ 2 = H_ {c2} / H_ {cm}$. (The introduced by Ginzburg and Landau in the original work is $\kappa _ {GL} = \kappa /\sqrt{2}$). In the approximation of the Ginzburg-Landau functional, $\kappa$ is temperature independent. For $\kappa = 1$ all three critical fields $H_ {c1}$, $H_ {c2}$ and $H_ {cm}$ coincide. Multi-component superconductors may show much more broad spectrum of states in an external magnetic field. Magnetic fields $H_{cm}$ and $H_{c2}$ are quite easy to calculate. However, in order to identify the state in an external magnetic field, we need to find also $H_{c1}$. As a result, in addition to the unusual sequence of the critical fields, the possibility of overscreening can be realized. In this case, it becomes possible for the jump-like transition between different solutions of the Abrikosov lattices. The calculation of the critical field $H_{c1}$ is again given by the solution of the set of Eqs. (69-74) which explicitly take into account the relation between the phases $\tilde{\phi }_1$ and $\tilde{\phi }_2$, which is imposed by the equation $div \textbf{j} = 0$. Instead of one singular point $\kappa =1$ existing in single-component superconductors, in a multicomponent superconductor in any case four parameters ($\theta ,\theta _1$,$(b_1-b_2)$,$ln(T_{c1}/T_{c2})$) form basis for criterion set of singular “surfaces.” Investigation of physical states with parameters close to this set present special large interest and can be made inside presented method.

## Appendix A: Dimensionless Form of the Equations

For numerical calculations it is convenient to bring the equations to a dimensionless form. For these purposes, we use the following substitutions:

$$\begin{aligned} \rho =&\; \gamma t_0 \text{ with } \gamma ^2 = \frac{\hbar ^2}{2m_1},\,\,\,|\Psi _1| = |\tilde{\Psi }_1| |\Psi _1|_{inf}, \\& |\Psi _2| = |\tilde{\Psi }_2| |\Psi _2|_{inf}, \,\,\,\, A_\phi = \frac{\hbar c}{2e\gamma } \tilde{A}. \end{aligned}$$

Here $|\Psi _1|_{inf}$ and $|\Psi _2|_{inf}$ are values of $|\Psi _1|$ and $|\Psi _2|$ at $\rho \rightarrow \infty$. They can be obtained from Eqs. (30-32):

44 $$\begin{aligned} \begin{aligned} |\Psi _1|^2_{inf}&= \frac{1}{b_1 b_2} \left\{ (b_1 \sin ^2 \theta _1 + b_2 \cos ^2\theta _2 )\left[ \cos ^2\theta \ln \left( \frac{T_{c1}}{T}\right) + \sin ^2\theta \ln \left( \frac{T_{c2}}{T}\right) \right] \right. \\&- \left. \frac{1}{2} \sin (2\theta _1) (b_1 - b_2 ) \left[ \sin ^2\theta \ln \left( \frac{T_{c1}}{T}\right) + \cos ^2\theta \ln \left( \frac{T_{c2}}{T}\right) \right] \right\} , \end{aligned} \end{aligned}$$

45 $$\begin{aligned} \begin{aligned} |\Psi _2|^2_{inf}&= \frac{1}{b_1b_2} \left\{ (b_1 \cos ^2 \theta _1 + b_2 \sin ^2\theta _2 )\left[ \sin ^2\theta \ln \left( \frac{T_{c1}}{T}\right) + \cos ^2\theta \ln \left( \frac{T_{c2}}{T}\right) \right] \right. \\&- \left. \frac{1}{2} \sin (2\theta _1) (b_1 - b_2 ) \left[ \cos ^2\theta \ln \left( \frac{T_{c1}}{T}\right) + \sin ^2\theta \ln \left( \frac{T_{c2}}{T}\right) \right] \right\} \end{aligned} \end{aligned}$$

and

46 $$\begin{aligned} cos(\mu _{inf})= \frac{\sin (2\theta ) \ln \left( T_{c1}/T_{c2}\right) }{2|\Psi _1|_{inf} |\Psi _2|_{inf}\sin (2\theta _1)(b_1-b_2)}. \end{aligned}$$

Then, Eqs. (14, 15) take the following form:

47 $$\begin{aligned} \begin{aligned}&-\left( \frac{1}{t_0} \frac{\partial |\tilde{\Psi }_1|}{\partial t_0} + \frac{\partial ^2 |\tilde{\Psi }_1|}{\partial t_0^2} \right) + \left( \Gamma ^2 \left( \frac{\partial \mu }{\partial t_0} \right) ^2 + \frac{1}{t_0^2} (1 -\tilde{A}t_0)^2\right) |\tilde{\Psi }_1| \\ {}&+ 2 |\Psi _1|^2_{inf} (b_1 \cos ^2 \theta _1 + b_2 \sin ^2 \theta _2) |\tilde{\Psi }_1|^3 - |\tilde{\Psi }_1| |\tilde{\Psi }_2|^2 \sin (2\theta _1) |\Psi _2|_{inf}^2 (b_1 - b_2) \cos (2 \mu ) \\&- 2 |\tilde{\Psi }_1| \left( \cos ^2 \theta \ln \left( \frac{T_{c1}}{T}\right) + \sin ^2 \theta \ln \left( \frac{T_{c2}}{T} \right) \right) + \sin (2\theta ) \frac{|\Psi _2|_{inf}}{|\Psi _1|_{inf}} |\tilde{\Psi }_2| \ln \left( \frac{T_{c1}}{T_{c2}}\right) \cos \mu = 0 \end{aligned} \end{aligned}$$

and

48 $$\begin{aligned} \begin{aligned}&-\frac{m_1}{m_2}\left( \frac{1}{t_0} \frac{\partial |\tilde{\Psi }_2|}{\partial t_0} + \frac{\partial ^2 |\tilde{\Psi }_2|}{\partial t_0^2} \right) + \frac{m_1}{m_2} \left( (\Gamma -1)^2 \left( \frac{\partial \mu }{\partial t_0} \right) ^2 + \frac{1}{t_0^2} (1 -\tilde{A}t_0)^2\right) |\tilde{\Psi }_2| \\&+ 2 |\Psi _2|^2_{inf} (b_1 \sin ^2 \theta _1 + b_2 \cos ^2 \theta _2) |\tilde{\Psi }_2|^3 - |\tilde{\Psi }_2| |\tilde{\Psi }_1|^2 \sin (2\theta _1) |\Psi _1|_{inf}^2 (b_1 - b_2) \cos (2 \mu ) \\&- 2 |\tilde{\Psi }_2| \left( \sin ^2 \theta \ln \left( \frac{T_{c1}}{T}\right) + \cos ^2 \theta \ln \left( \frac{T_{c2}}{T} \right) \right) + \sin (2\theta ) \frac{|\Psi _1|_{inf}}{|\Psi _2|_{inf}} |\tilde{\Psi }_1| \ln \left( \frac{T_{c1}}{T_{c2}}\right) \cos \mu = 0. \end{aligned} \end{aligned}$$

Here $$\Gamma$$ is:

49 $$\begin{aligned} \Gamma = \left( 1 + \frac{m_2}{m_1} \frac{|\tilde{\Psi }_1|^2|\Psi _1|_{inf}^2}{|\tilde{\Psi }_2|^2|\Psi _2|_{inf}^2}\right) ^{-1}. \end{aligned}$$

The Maxwell equation in the dimensionless variable has the following form:

50 $$\begin{aligned} - \left( \frac{1}{t_0}\frac{\partial \tilde{A}}{\partial t_0} + \frac{\partial ^2 \tilde{A}}{\partial t_0^2} \right) + \frac{8\pi e^2 \gamma ^2}{m_1 c^2} |\Psi _1|_{inf}^2 \left( |\tilde{\Psi }_1|^2+ \frac{m_1}{m_2} \frac{(|\Psi _2|_{inf})^2}{(|\Psi _1|_{inf})^2} |\tilde{\Psi }_2|^2 \right) \left( \tilde{A} - \frac{1}{t_0}\right) +\frac{1}{t_0^2} \tilde{A} = 0 \end{aligned}$$

The equation for $\mu$ is

51 $$\begin{aligned} \begin{aligned}&- \frac{1}{t_0} \frac{\partial }{\partial t_0 } \left( t_0 |\tilde{\Psi }_1|^2 \Gamma ^2 \frac{\partial \mu }{\partial t_0}\right) - \frac{m_1}{m_2} \frac{|\Psi _2|_{inf}^2}{|\Psi _1|_{inf}^2}\frac{1}{t_0}\frac{\partial }{\partial t_0} \left( t_0 |\tilde{\Psi }_2|^2(1 - \Gamma )^2 \frac{\partial \mu }{\partial t_0} \right) \\&+\sin (2 \theta _1) |\Psi _2|_{inf}^2 |\tilde{\Psi }_1|^2 |\tilde{\Psi }_2|^2 (b_1 - b_2) \sin (2 \mu ) - \sin (2\theta ) \frac{|\Psi _2|_{inf}}{|\Psi _1|_{inf}} |\tilde{\Psi }_1| |\tilde{\Psi }_2| \ln \left( \frac{T_{c1}}{T_{c2}}\right) \sin \mu =0, \end{aligned} \end{aligned}$$

The magnetic field *H* is equal to

$$\begin{aligned} H = \frac{\hbar c}{2 e \gamma ^2} \tilde{H}, \tilde{H} = \frac{\partial \tilde{A}}{\partial t_0 } + \frac{\tilde{A}}{t_0}. \end{aligned}$$

## Appendix B: Approximation in the Range $t_0 \ll 1$.

In the range $t_0 \ll 1$ the functions $|\tilde{\Psi }_1|$, $|\tilde{\Psi }_2|$ are odd functions of $t_0$, while the function $\mu$ is an even function of $t_0$. They can be expanded in this range as:

52 $$\begin{aligned} \begin{aligned} |\tilde{\Psi }_1| = \alpha _1 t_0 - \alpha _3 t_0^3 + \alpha _5t_0^5+... |\tilde{\Psi }_2| = \beta _1 t_0 - \beta _3 t_0^3 + \beta _5t_0^5+... \end{aligned} \end{aligned}$$

53 $$\begin{aligned} \begin{aligned} \tilde{A} = a_1 t_0 - a_3 t_0^3 + a_5t_0^5+... \mu = c_0 - c_2 t_0^2 + c_4t_0^4+... \end{aligned} \end{aligned}$$

The coefficients $\{ \alpha _1, \beta _1, a_1, c_0\}$ can be found from the boundary conditions at $t_0 \rightarrow \infty$ see Eqs. (44-46). Inserting the expansions Eqs. (52 and 53) into Eqs. (47-51) we obtain the next expression for the coefficients $\{\alpha _3,\alpha _5,\beta _3,\beta _5,a_3,a_5,c_2,c_4,\Gamma _0 \}$, $\Gamma =\Gamma _0 + \Gamma _1 t_0^2$:

$$\begin{aligned} \Gamma _0 = \left( 1 + \frac{m_2}{m_1} \frac{\alpha _1^2}{\beta _1^2} \frac{|\Psi _1|_{inf}^2}{|\Psi _2|_{inf}^2}\right) ^{-1} \end{aligned}$$

$$\begin{aligned} \Gamma _1 = 2 \frac{m_2}{m_1} \frac{\alpha _1^2}{ \beta _1^2} \frac{|\Psi _1|_{inf}^2}{|\Psi _2|_{inf}^2} \left( \frac{\alpha _3}{\alpha _1} - \frac{\beta _3}{\beta _1} \right) \Gamma _0^2 \end{aligned}$$

Correspondingly, we get the following set of equations for the expansion coefficients from Eq. (47):

54 $$\begin{aligned} 8 \alpha _3 &- 2 \alpha _1 a_1 - 2 \alpha _1 \left( \cos ^2 \theta \ln \left( \frac{T_{c1}}{T}\right) +\sin ^2 \theta \ln \left( \frac{T_{c2}}{T}\right) \right) \\&+ \beta _1\sin (2 \theta ) \frac{|\Psi _2|_{inf}}{|\Psi _1|_{inf}} \ln \left( \frac{T_{c1}}{T_{c2}} \right) \cos c_0 = 0 \end{aligned}$$

and

55 $$\begin{aligned} \begin{aligned}&-24 \alpha _5 + 4 \alpha _1 c_2^2 \Gamma _0^2 + 2 (\alpha _3 a_1 + \alpha _1 a_3) + \alpha _1 a^2_1 +2 |\Psi _1|_{inf}^2 (b_1 \cos ^2\theta _1 + b_2 \sin ^2\theta _1 ) \alpha _1^3 \\&+ \sin (2\theta _1) \alpha _1 \beta _1^2 |\Psi _2|_{inf}^2 (b_1 - b_2) (1 - 2 \cos ^2 (c_0)) + 2 \alpha _3 \left( \cos ^2 \theta \ln \left( \frac{T_{c1}}{T} \right) + \sin ^2 \theta \ln \left( \frac{T_{c2}}{T}\right) \right) \\&+\sin (2 \theta ) \frac{|\Psi _2|_{inf}}{|\Psi _1|_{inf}} \ln \left( \frac{T_{c1}}{T_{c2}}\right) (-\beta _3 \cos (c_0) + \beta _1 c_2 \sin (c_0)) = 0, \end{aligned} \end{aligned}$$

and from Eq. (48):

56 $$\begin{aligned} \frac{m_1}{m_2} &\;(8 \beta _3 - 2 \beta _1a_1 ) - 2\beta _1\left( \cos ^2 \theta \ln \left( \frac{T_{c2}}{T} \right) + \sin ^2 \theta \ln \left( \frac{T_{c1}}{T}\right) \right) \\&+ \alpha _1\sin (2\theta ) \frac{|\Psi _1|_{inf}}{|\Psi _2|_{inf}} \ln \left( \frac{T_{c1}}{T_{c2}}\right) \cos ( c_0) = 0 \end{aligned}$$

and

57 $$\begin{aligned} \begin{aligned} \frac{m_1}{m_2} [ -24 \beta _5&+4 \beta _1 c_2^2(1 - \Gamma _0)^2 + 2 (\beta _1 a_3 + \beta _3 a_1) + \beta _1 a_1^2 ] + 2 |\Psi _2|_{inf}^2 (b_1 \sin ^2 \theta _1 + b_2 \cos ^2 \theta _1) \beta _1^3 \\ {}&+ \sin (2\theta _1 ) \beta _1 \alpha _1^2 |\Psi _1|^2_{inf} (b_1 - b_2) (1- 2 \cos ^2 c_0) + 2 \beta _3 \left( \cos ^2 \theta \ln \left( \frac{T_{c2}}{T} \right) + \sin ^2 \theta \ln \left( \frac{T_{c1}}{T}\right) \right) \\ {}&+ \sin (2\theta ) \frac{|\Psi _1|_{inf}}{|\Psi _2|_{inf}} \ln \left( \frac{T_{c1}}{T_{c2}}\right) (\alpha _1 c_2 \sin (c_0) - \alpha _3 \cos (c_0)) = 0. \end{aligned} \end{aligned}$$

Further, the Maxwell equation gives the following set of equations:

58 $$\begin{aligned} 8 a_3 - \frac{8\pi e^2 \gamma ^2 }{m_1 c^2} |\Psi _1|^2_{inf} \left( \alpha _1^2 +\frac{m_1}{m_2} \frac{|\Psi _2|_{inf}^2}{|\Psi _1|_{inf}^2} \beta _1^2\right) =0 \end{aligned}$$

and

59 $$\begin{aligned} -24 a_5 + \frac{8\pi e^2 \gamma ^2}{m_1 c^2} |\Psi _1|^2_{inf} \left[ a_1 \left( \alpha _1^2 +\frac{m_1}{m_2} \frac{|\Psi _2|_{inf}^2}{|\Psi _1|_{inf}^2} \beta _1^2\right) \right. + \left. 2 \left( \alpha _1 \alpha _3 + \frac{m_1}{m_2} \frac{|\Psi _2|_{inf}^2}{|\Psi _1|_{inf}^2} \beta _1 \beta _3 \right) \right] = 0. \end{aligned}$$

And the equation for $\mu$ yields:

60 $$\begin{aligned} 8 &\;\alpha _1^2 \Gamma _0^2 c_2 + 8 \frac{m_1}{m_2} \frac{|\Psi _2|_{inf}^2}{|\Psi _1|_{inf}^2} \beta _1^2 (1 - \Gamma _0 )^2 c_2 \\&- \sin (2 \theta ) \frac{|\Psi _2|_{inf}}{|\Psi _1|_{inf}} \ln \left( \frac{T_{c1}}{T_{c2}}\right) \alpha _1 \beta _1 \sin ( c_0) = 0 \end{aligned}$$

61 $$\begin{aligned} \begin{aligned}&-24 \left[ \alpha _1 \alpha _3 \Gamma _0^2 c_2 - \alpha _1^2 \Gamma _0\Gamma _1 c_2 +\alpha _1^2\Gamma _0^2 c_4 \right] \\ {}&- 24\frac{m_1}{m_2} \frac{|\Psi _2|_{inf}^2}{|\Psi _1|_{inf}^2}(\beta _1\beta _3(1 - \Gamma _0)^2 c_2 + (1-\Gamma _0)\Gamma _1 \beta _1^2 c_2 + c_4 \beta _1^2 (1 - \Gamma _0)^2 ) \\&+ \sin (2\theta _1) |\Psi _2|_{inf}^2 \alpha _1^2 \beta _1^2 (b_1 - b_2) \sin (2c_0) + \sin (2\theta ) \frac{|\Psi _2|_{inf}}{|\Psi _1|_{inf}} \ln \left( \frac{T_{c1}}{T_{c2}}\right) \\ {}&\times ( (\alpha _3\beta _1 + \alpha _1 \beta _3 )\sin (c_0) + c_2 \cos (c_0) \alpha _1 \beta _1) =0 \end{aligned} \end{aligned}$$

## Appendix C. Numerical Solution at $\rho = \infty$.

For parameter values, given by Eq. (28), we obtain the next values for quantities $\{ |\Psi _1|^2_{inf},|\Psi _2|^2_{inf}, \cos (\mu _{inf})\}$.

62 $$\begin{aligned} |\Psi _1|_{inf}^2 = \left\{ \begin{array}{ll} 1.209457\cdot 10^{4} ; &{} \theta = 0\\ {1.205556\cdot 10^{4}} ; &{} \theta = 0.1 \\ 1.175276\cdot 10^{4} ; &{} \theta = 0.3 \end{array}\right| _{Gauss^2} \end{aligned}$$

63 $$\begin{aligned} |\Psi _2|_{inf}^2 = \left\{ \begin{array}{ll} 6.464209\cdot 10^{3} ; &{} \theta = 0\\ 6.487598\cdot 10^{3} ; &{} \theta = 0.1 \\ 6.669154\cdot 10^{3} ; &{} \theta = 0.3 \end{array}\right| _{Gauss^2} \end{aligned}$$

64 $$\begin{aligned} \cos (\mu _{inf}) = \left\{ \begin{array}{ll} 0 ; &{} \theta = 0\\ { -0.0774303} ; &{} \theta = 0.1\\ -0.2198284 ; &{} \theta = 0.3 \end{array}\right. \end{aligned}$$

Further, for the numerical calculations, we will use

65 $$\begin{aligned} \frac{8\pi e^2 \gamma ^2 }{m_1 c^2} = 3.573832\cdot 10^{-5} (Gauss)^{-2} \end{aligned}$$

## Appendix D. Numerical Solution for $\theta =0$

In the range of parameter $t_0\gg 1$, we have the following asymptotic behavior of $\tilde{A}$ and $\tilde{H}$:

66 $$\begin{aligned} \tilde{A} \approx \frac{1}{t_0} +\frac{R}{\sqrt{t_0}}e^{-t_0/\lambda } \left( 1+ \frac{3\lambda }{8t_0} - \frac{15 \lambda ^2}{128 t_0^2 } \right) \end{aligned}$$

and

$$\begin{aligned} \tilde{H} \approx - \frac{R}{\lambda \sqrt{t_0}} \left( 1 - \frac{\lambda }{8 t_0} + \frac{9 \lambda }{128 t_0^2} \right) e^{- t_0/\tilde{\lambda }} \end{aligned}$$

with

$$\begin{aligned} \tilde{\lambda }^{-2} = \frac{8\pi e^2 \gamma ^2 }{c^2 m_1} \left( |\Psi _1|^2_{inf} + |\Psi _2|^2_{inf} \frac{m_1}{m_2}\right) . \end{aligned}$$

For $\theta = 0$, we get $\tilde{\lambda }^{-2} = 0.894279$. The asymptotics for $|\tilde{\Psi _{1}}|$ and $|\tilde{\Psi _{2}}|$ have the form:

67 $$\begin{aligned} |\tilde{\Psi }_1| =&\; 1- \frac{S_{11}}{\sqrt{t_0} }\exp (-\kappa _1 t_0) \left( 1 - \frac{1}{8\kappa _1t_0}\right) \\& - \frac{S_{12}}{\sqrt{t_0}} \exp (-\kappa _2 t_0)\left( 1-\frac{1 }{8\kappa _2t_0}\right) \end{aligned}$$

68 $$\begin{aligned} |\tilde{\Psi }_2| =&\; 1- \frac{S_{21}}{\sqrt{t_0} }\exp (-\kappa _1 t_0) \left( 1 - \frac{1}{8\kappa _1t_0}\right) \\&- \frac{S_{22}}{\sqrt{t_0}} \exp (-\kappa _2 t_0)\left( 1-\frac{1 }{8\kappa _2t_0}\right) \end{aligned}$$

with $S_{21}/S_{11} = 3.62365$, $S_{22}/S_{12} =- 0.258166$. Here quantities $\{R, S_{11}, S_{12}\}$ are some constants, which can be found by solving the full set of the differential equations. In the range $t_0 \ll 1$, we obtain from Eqs. (58-61).

69 $$\begin{aligned} \alpha _3 = 0.25 \alpha _1 a_1 + 0.04558 \alpha _1 \end{aligned}$$

70 $$\begin{aligned} \beta _3 = 0.25 \beta _1 a_1 + 0.0119138 \beta _1 \end{aligned}$$

71 $$\begin{aligned} a_3 = 0.05403(\alpha _1^2 + 1.068944\beta _1^2) \end{aligned}$$

72 $$\begin{aligned} a_5 = 0.01801 \{ a_1 (\alpha _1^2 +1.06894 \beta _1^2) + 2(\alpha _1\alpha _3 + 1.068944 \beta _1\beta _3)\} \end{aligned}$$

73 $$\begin{aligned} \alpha _5 = \frac{1}{24} \left\{ 2(\alpha _1 \alpha _3 +\alpha _1 a_3) +\alpha _1 a_1^2 +0.446235\alpha _1^3 - 0.0815917 \alpha _1 \beta _1^2 + 0.364643 \alpha _3 \right\} \end{aligned}$$

74 $$\begin{aligned} \beta _5 = \frac{1}{24} \left\{ 2 (\beta _1 a_3+\beta _3a_1)+\beta _1a_1^2 +0.171639\beta _1^3 - 0.0763292\beta _1 \alpha _1^2 + 0.09531 \beta _3\right\} . \end{aligned}$$

For small $\theta$ the function $\mu$ can be presented in the form $\mu = \pi /2 + \delta$ with $\delta \propto \theta \ll 1$. So, for $\theta =0.1$ Eq. (A.8) in the first order of perturbation theory over $\theta$ can be presented in the form:

75 $$\begin{aligned} \begin{aligned}&-\frac{1}{t_0} \frac{\partial }{\partial t_0} \left( t_0 |\tilde{\Psi }_1|^2 \Gamma ^2 \frac{\partial \delta }{\partial t_0} \right) - 1.07628 \frac{1}{t_0} \frac{\partial }{\partial t_0} \left( t_0 |\tilde{\Psi }_2|^2 (1-\Gamma )^2 \frac{\partial \delta }{\partial t_0} \right) \\ {}&+0.163774 |\tilde{\Psi }_1|^2|\tilde{\Psi }_2|^2\delta = 1.268103\cdot 10^{-2} |\tilde{\Psi }_1||\tilde{\Psi }_2| \end{aligned} \end{aligned}$$

Corrections to quantities $\{|\tilde{\Psi }_1|, |\tilde{\Psi }_2| \}$ are of the second order by $\theta$. Hence, in the leading approximation, we can use the values of function $\{|\tilde{\Psi }_1|, |\tilde{\Psi }_2|\}$ at the point $\theta =0$.

## Appendix E. Numerical Solution of the Eqs. $\theta =0$

It is follows from Eq. (46) the point $\theta =0$ is singular. It this point $\mu = \pm \pi /2$. As the result the equation system from four equations Eqs. (47-50) reduces to the system of three equations. The solution of its has a special interest, since the solution is more simple in such case and can be easy spread on a large region over $\theta$. Solving Eqs. (44-46) on estimates the four parameters $\left\{ \alpha _1, \beta _1, a_1, c_0 \right\}$. Their values are presented in the table.

**Table 1:** Resulting values of the expansion at $t_0\ll 1$ for $\alpha _1$, $\beta _1$, $a_1$ and $c_0$ and the critical magnetic fields $H_{c1}$, $H_{c2}$ and $H_{c}$

	$\alpha _1$	$\beta _1$	$a_1$	$c_0$	$\tilde{H}(0)$	$\tilde{H}_{c1}$	$\tilde{H}_{c}$	$H_{c2}$
$\theta =0$	0.502624	0.381376	0.178348	$\pm \pi /2$	0.356697	0.381862	0.31753	0.36464
$\theta =0.3$	0.500653	0.390095	0.179133	$\pi /2 + 0.321145$	0.358285	0.383238	0.318328	0.354247
$\theta =0.6$	0.491193	0.406449	0.179380	$\pi /2 + 0.528922$	0.359888	0.385749	0.317728	0.325411
$\theta =0.9$	0.468440	0.416228	0.178354	$\pi /2 + 0.560478$	0.356357	0.386459	0.311181	0.284332

At $\theta = 0$, we have the next equation for $\{|\tilde{\Psi }_1|,|\tilde{\Psi }_2|, \tilde{A}\}$

76 $$\begin{aligned} \begin{aligned} - \left( \frac{1}{t_0} \frac{\partial |\tilde{\Psi }_1|}{\partial t_0}+ \frac{\partial ^2|\tilde{\Psi }_1|}{\partial t_0^2} \right)&+ \frac{1}{t_0^2}(1- \tilde{A} t_0)^2 |\tilde{\Psi }_1| + 0.446235 |\Psi _1|^3 \\&{ -} 0.0815917 |\tilde{\Psi }_1||\tilde{\Psi }_2|^2 - 0.364643 |\tilde{\Psi }_1| = 0 \end{aligned} \end{aligned}$$

77 $$\begin{aligned} \begin{aligned} - 2\left( \frac{1}{t_0} \frac{\partial |\tilde{\Psi }_2|}{\partial t_0}+ \frac{\partial ^2|\tilde{\Psi }_2|}{\partial t_0^2} \right)&+ \frac{2}{t_0^2}(1- \tilde{A} t_0)^2 |\tilde{\Psi }_2| + 0.343279 |\Psi _2|^3 \\&{-} 0.152658 |\tilde{\Psi }_2||\tilde{\Psi }_1|^2 - 0.1906204 |\tilde{\Psi }_2| = 0 \end{aligned} \end{aligned}$$

78 $$\begin{aligned} \begin{aligned} - \left( \frac{1}{t_0} \frac{\partial \tilde{A}}{\partial t_0}+ \frac{\partial ^2\tilde{A}}{\partial t_0^2} \right) + 0.43224(|\tilde{\Psi }_1|^2 + 1.068944|\tilde{\Psi }_2|^2)\left( \tilde{A} - \frac{1}{t_0}\right) +\frac{1}{t_0^2} \tilde{A} = 0 \end{aligned} \end{aligned}$$

From the numerical solution, we find the coefficients *R*, $S_{11}$ and $S_{22}$ in asymptotics presented by Eqs. (66-68):

79 $$\begin{aligned} R=- 4.89675 , S_{11} = 3.32825 , S_{22} = 2.33331. \end{aligned}$$

## Appendix F. Small $\theta$ Values, Correction to the Phase Difference

We obtain the next equation for the function $\delta (t_0)$ in the region $t_0\ll 1$.

80 $$\begin{aligned} \delta = \delta _0 - \delta ^{(2)} t_0^2 + \delta ^{(4)} t_0^4 \end{aligned}$$

where

$$\begin{aligned} \alpha _3 = 5.15 \cdot 10^{-2}, \beta _3 = 2.60634 \cdot 10^{-2}, \Gamma _0 = 0.392089 , \Gamma _1 =1.58942 \cdot 10^{-2} , \end{aligned}$$

81 $$\begin{aligned} \delta ^{(2)} = 1.585135\cdot 10^{-3} \frac{\alpha _1 \beta _1}{\alpha _1^2 \Gamma _0^2 + 1.0762888\beta _1^2 (1-\Gamma _0)^2 }= 3.129603\cdot 10^{-3} \end{aligned}$$

$$\begin{aligned} \delta ^{(4)} = -7.180505\cdot 10^{-5} + 2.982978\cdot 10^{-3} \delta _0 \end{aligned}$$

Numerical calculations for $\theta = 0.1$ give

82 $$\begin{aligned} \delta _{inf} = 0.077430178, \delta _0=0.111235. \end{aligned}$$

For $t_0 \gg 1$, we have the following asymptotic $\delta \approx \delta _{inf} + \frac{0.2917}{\sqrt{t_0}} e^{-0.5620824 t_0}$. The phase difference in full range of $t_0$ for $\theta = 0.1$ is presented at Fig. 3.

## Appendix G. Case $\theta = 0.3$

Consider now the case of $\theta =0.3$. Parameters $\{\alpha _1, \beta _1, a_1, c_0 \}$ are free parameters and for quantities $\left\{ \alpha _3, \beta _3, a_3, \alpha _5, \beta _5, a_5, c_2, c_4\right\}$, we obtain from Eqs. (52-57) in the region $t_0 \ll 1$ the following values:

$$\begin{aligned} \alpha _3 = 0.25 \alpha _1 a_1 + 4.3680665 \cdot 10^{-2} \alpha _1 - 4.744229\cdot 10^{-3} \beta _1 \cos (c_0) \end{aligned}$$

$$\begin{aligned} \beta _3 = 0.25 \beta _1 a_1 + 1.286363 \cdot 10^{-2} \beta _1 - 3.974876\cdot 10^{-3} \alpha _1 \cos (c_0) \end{aligned}$$

$$\begin{aligned} \Gamma _0 = (1+0.837834\cdot \alpha _1^2/\beta _1^2)^{-1}, \Gamma _1 = \frac{1.675668 \alpha _1^2}{\beta _1^2} \Gamma _0^2 \left( \frac{\alpha _3}{\alpha _1} - \frac{\beta _3}{\beta _1}\right) \end{aligned}$$

$$\begin{aligned} c_2 = \frac{4.744229\cdot 10^{-3} \alpha _1\beta _1\sin (c_0) }{\alpha _1 \Gamma _0^2 + 1.193554 \beta _1^2(1- \Gamma _0)^2} \end{aligned}$$

$$\begin{aligned} \begin{aligned} a_3&= 4.992322\cdot 10^{-2} (\alpha _1^2 + 1.193554\beta _1^2) \\ \alpha _5&= \frac{1}{24} \left\{ 4\Gamma _0^2 \alpha _1 c_2^2 + 2 (\alpha _1 a_3 + \alpha _3 a_1) + \alpha _1 a_1^2 \right. + \left. 0.412317\alpha _1^3 - 8.417844\cdot 10^{-2} \alpha _1 \beta _1^2 (1 -2\cos ^2 (c_0)) + 0.349445 \alpha _3 \right. \\&+ \left. 3.79538\cdot 10^{-2} (-\beta _3 \cos (c_0) + \beta _1c_2 \sin (c_0) ) \right\} \\ \beta _5&= \frac{1}{24} \left\{ 4(1-\Gamma _0)^2 \beta _1 c_1^2 + 2 (\beta _1 a_3 + \beta _3 a_1) + \beta _1 a_1^2 + 0.177081\beta _1^3 - 7.052755\cdot 10^{-2} \alpha _1^2 \beta _1 (1 -2\cos ^2 (c_0)) \right. \\&+ \left. 0.10290903 \beta _3 + 3.1799\cdot (-\alpha _3 \cos (c_0) + \alpha _1 c_2 \sin (c_0) ) \right\} \\ a_5&= 1.664107\cdot 10^{-2}\{ a_1 (\alpha _1^2 + 1.193554\beta _1^2)+2(\alpha _1 \alpha _3 + 1.193554 \beta _1\beta _3)\} \\ c_4&= \frac{1}{\alpha _1^2 \Gamma _0^2 + 1.193554 \beta _1^2 (1 - \Gamma _0)^2 } \{ (\alpha _1^2\Gamma _0\Gamma _1 - \alpha _1 \alpha _3 \Gamma _0^2)c_2 -1.193554(\beta _1\beta _3(1 -\Gamma _0)^2 \\&+(1-\Gamma _0) \Gamma _1 \beta _1^2) c_2 - 3.507435\cdot 10^{-3} \alpha _1^2 \beta _1^2\sin (2c_0) + 1.58141\cdot 10^{-3} (\alpha _3\beta _1 \sin c_0 + \alpha _1 \beta _3 \sin c_0 \\&+ c_2 \alpha _1\beta _1 \cos c_0)\} \end{aligned} \end{aligned}$$

At $t_0\rightarrow \infty$ the variable tends to $\mu _{inf} \rightarrow \pi /2 + 0.2216385$ and $\Gamma _{\inf }\rightarrow 0.531596$.

For $\theta = 0.3$, we obtain the following system of differential equations for quantities $\{ |\tilde{\Psi }_1|,|\tilde{\Psi }_2|,\tilde{A}, \mu \}$:

83 $$\begin{aligned} \begin{aligned}&-\left( \frac{1}{t_0} \frac{\partial |\tilde{\Psi }_1|}{\partial t_0} + \frac{\partial ^2 |\tilde{\Psi }_1|}{\partial t_0} \right) + \left( \Gamma ^2 \left( \frac{\partial \mu }{\partial t_0} \right) ^2 + \frac{1}{t_0^2} (1 -\tilde{A}t_0)^2\right) |\tilde{\Psi }_1| + B_{13} |\tilde{\Psi }_1|^3 + B_{11} |\tilde{\Psi }_1| \\ {}&+ C_{13} |\tilde{\Psi }_1| |\tilde{\Psi }_2|^2 (1 - 2 \cos ^2 \mu ) + C_{11} |\tilde{\Psi }_2|\cos \mu = 0 \end{aligned} \end{aligned}$$

84 $$\begin{aligned} \begin{aligned}&-\left( \frac{1}{t_0} \frac{\partial |\tilde{\Psi }_2|}{\partial t_0} + \frac{\partial ^2 |\tilde{\Psi }_1|}{\partial t_0} \right) + \left( (1-\Gamma )^2 \left( \frac{\partial \mu }{\partial t_0} \right) ^2 + \frac{1}{t_0^2} (1 -\tilde{A}t_0)^2\right) |\tilde{\Psi }_2| + B_{23} |\tilde{\Psi }_2|^3 +B_{21}|\tilde{\Psi }_2| \\&+ C_{23} |\tilde{\Psi }_2||\tilde{\Psi }_1|^2(1-2\cos ^2\mu ) +C_{21} |\tilde{\Psi }_1| \cos \mu = 0 \end{aligned} \end{aligned}$$

85 $$\begin{aligned} - \frac{1}{t_0} \frac{\partial }{\partial t_0} \left( t_0\frac{\partial \tilde{A}}{\partial t_0}\right) + \left( F_1|\tilde{\Psi }_1|^2 + F_2 |\tilde{\Psi }_2|^2\right) \left( \tilde{A} - \frac{1}{t_0}\right) + \frac{1}{t_0^2 } \tilde{A}=0 \end{aligned}$$

86 $$\begin{aligned} \begin{aligned}&-\frac{1}{t_0} \frac{\partial }{\partial t_0} \left( t_0 |\tilde{\Psi }_1|^2 \Gamma ^2 \frac{\partial \mu }{\partial t_0}\right) - G_0\frac{1}{t_0} \frac{\partial }{\partial t_0} \left( t_0 |\tilde{\Psi }_2|^2 (1-\Gamma )^2 \frac{\partial \mu }{\partial t_0} \right) -C_{13}|\tilde{\Psi }_1|^2 |\tilde{\Psi }_2|^2 \sin (2\mu ) \\&-C_{11}\cdot |\tilde{\Psi }_1| |\tilde{\Psi }_2| \sin \mu =0, \end{aligned} \end{aligned}$$

where

87 $$\begin{aligned} \Gamma = (1 + 0.881129 |\tilde{\Psi }_1|^2/|\tilde{\Psi }_2|^2)^{-1} \end{aligned}$$

and

$$\begin{aligned} B_{11}= - 0.349445, \, B_{13} = 0.433624, \, C_{13} = - 8.417844\cdot 10^{-2}, \, C_{11} = 3.700964 \cdot 10^{-2}, \end{aligned}$$

$$\begin{aligned} B_{21}= - 0.102909, \, B_{23} = 0.177081, \, C_{23} = -7.4172086\cdot 10^{-2}, \, C_{21} = 3.261003\cdot 10^{-2}, \end{aligned}$$

$$\begin{aligned} F_1 = 0.420024, \, F_2 = 0.476689 , \, G_0 =1.134908. \end{aligned}$$

At $\theta = 0.3$ at large distances $t_0\gg 1$, we get the following asymptotic expression for the magnetic field:

88 $$\begin{aligned} \tilde{ H}(t_0) = - \frac{2.82979}{\sqrt{t_0}} \left( 1- \frac{0.1320029}{t_0} \right) \exp \left( - 0.9469491t_0 \right) \end{aligned}$$

In both numerical investigated cases, the superconductor turns out unusual state. The value of $H_{c1}$ and $H_{c2}$ are larger that $H_{c}$.

The three correlation length can be estimated from the system of equations Eqs. (84-86):

89 $$\begin{aligned} \left( \begin{array}{ccc} 0.875383 -\kappa ^2 &{} -0.1602211 &{} 3.61043\times 10^{-2} \\ -0.141176 &{} 0.361331 - \kappa ^2 &{} 3.18117\times 10^{-2} \\ 6.791168\times 10^{-2} &{} 6.79117\times 10^{-2} &{}0.301396 - \kappa ^2 \end{array} \right) \left( \begin{array}{c} f_1 \\ f_2 \\ f_3 \end{array} \right) =0 \end{aligned}$$

The solution of the Eqs. (89) is

90 $$\begin{aligned} \begin{array}{l} \kappa _1 =0.50125, \textbf{f}_1 = (0.196846,0.541458, -1) \\ \kappa _2 =0.60715, \textbf{f}_2 = (0.183665,0.806245, 1) \\ \kappa _3 =0.95824, \textbf{f}_3 = (1,-0.248778, 8.27139\times 10^{-2}) \end{array} \end{aligned}$$

The numerical calculations of Eqs. (83-85) yields the following asymptotic expression for $\{|\tilde{\Psi }_1|,|\tilde{\Psi }_1|,\mu \}$:

91 $$\begin{aligned} \begin{array}{l} \left( \begin{array}{c} |\tilde{\Psi }_1|\\ |\tilde{\Psi }_2|\\ \mu \end{array} \right) = \left( \begin{array}{c} 1 \\ 1\\ \frac{\pi }{2}+0.2216385 \end{array} \right) - \frac{1.87027}{\sqrt{t_0}} \left( 1 - \frac{0.24938}{t_0} \right) \textbf{f}_1 \exp (-0.501254 t_0) \\ \\ -\frac{1.80889}{\sqrt{t_0}} \left( 1- \frac{0.20588}{t_0}\right) \textbf{f}_2\exp (-0.6071449t_0) -\frac{3.6315}{\sqrt{t_0}} \left( 1- \frac{0.13045}{t_0}\right) \textbf{f}_3\exp (-0.958238t_0) \end{array} \end{aligned}$$
